# Supplementary material for: Targeting mitochondrial dysfunction using methylene blue or mitoquinone to improve skeletal aging
Source: Aging (Albany NY). 2024 Mar 25;16(6):4948–64. doi: 10.18632/aging.205147 (PMC11006499; doi:10.18632/aging.205147)
Supplement: Supplementary Figure 1 [file aging-16-205147-s001.pdf]

SUPPLEMENTARY FIGURE

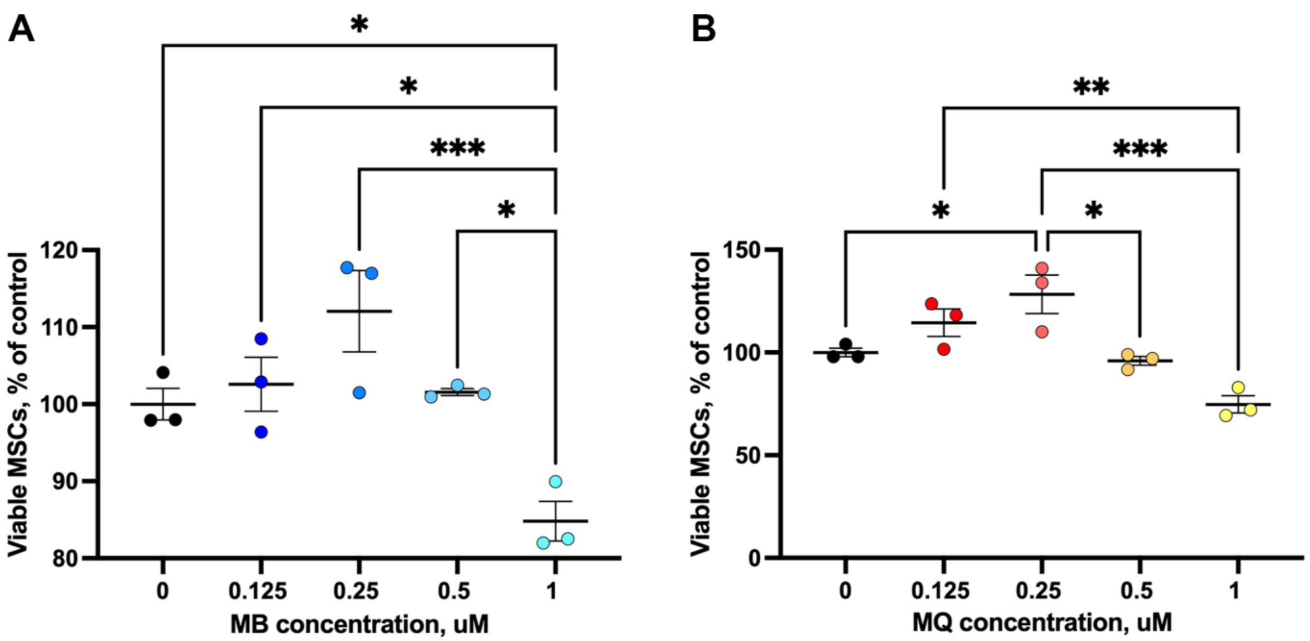

Supplementary Figure 1. Effects of MB or MitoQ on BMSCs viability. BMSCs isolated from 6–7 months were seeded on 96 wells plate in the presence or absence of MB (**A**) or MitoQ (**B**) (0, 0.125, 025, 0.5 uM and 1.0 uM) for 48 hours. Percentage of live cells was determined by calcein AM staining. Data presented as mean ± SEM of $n = 3$. Data tested by multivariate ANOVA. Significance accepted at $p < 0.05$ ($^{*}p < 0.05$, $^{**}p < 0.01$, $^{***}p < 0.001$, $^{****}p < 0.0001$).
